# miR-663a regulates growth of colon cancer cells, after administration of antimicrobial peptides, by targeting CXCR4-p21 pathway

**DOI:** 10.1186/s12885-016-3003-9

**Published:** 2017-01-07

**Authors:** Kengo Kuroda, Tomokazu Fukuda, Marija Krstic-Demonacos, Constantinos Demonacos, Kazuhiko Okumura, Hiroshi Isogai, Miwa Hayashi, Kazuki Saito, Emiko Isogai

**Affiliations:** 1 Laboratory of Animal Microbiology, Graduate School of Agricultural Science, Tohoku University, Sendai, 981-8555 Japan; 2 United Graduate School of Agricultural Sciences, Graduate School of Agricultural Science, Iwate University, Morioka, Iwate Japan; 3 School of Environment and Life Sciences, University of Salford, Salford, UK; 4 Division of Pharmacy and Optometry, Faculty of Biology Medicine and Health, School of Health Sciences, University of Manchester, Manchester, UK; 5 Department of Oral and Maxillofacial Surgery, School of Dentistry, Health Sciences University of Hokkaido, Hokkaido, Japan; 6 Animal Research Center, Sapporo Medical University, Sapporo, Japan

**Keywords:** miR-663a, HCT116 cells, Cationic Antimicrobial Peptides and CXCR4

## Abstract

**Background:**

Antimicrobial peptides (AMPs) play important roles in the innate immune system of all life forms and recently have been characterized as multifunctional peptides that have a variety of biological roles such as anticancer agents. However, detailed mechanism of antimicrobial peptides on cancer cells is still largely unknown.

**Methods:**

miRNA array and real-time qPCR were performed to reveal the behavior of miRNA in colon cancer HCT116 cells during the growth suppression induced by the AMPs. Establishment of miR-663a over-expressing HCT116 cells was carried out for the evaluation of growth both in vitro and in vivo. To identify the molecular mechanisms, we used western blotting analysis.

**Results:**

miR-663a is upregulated by administration of the human cathelicidin AMP, LL-37, and its analogue peptide, FF/CAP18, in the colon cancer cell line HCT116. Over-expression of miR-663a caused anti-proliferative effects both in vitro and in vivo. We also provide evidence supporting the view that these effects are attributed to suppression of the expression of the chemokine receptor CXCR4, resulting in the abrogation of phosphorylation of Akt and cell cycle arrest in G2/M via p21 activation.

**Conclusions:**

This study contributes to the understanding of the AMPs’ mediated anti-cancer mechanisms in colon cancer cells and highlights the possibility of using AMPs and miRNAs towards developing future strategies for cancer therapy.

**Electronic supplementary material:**

The online version of this article (doi:10.1186/s12885-016-3003-9) contains supplementary material, which is available to authorized users.

## Background

Colon cancer represents the third most commonly diagnosed malignancy in males and the second in females with more than 1.3 million cases reported and 693,900 deaths recorded in 2012 worldwide [1]. According to American Cancer Society, in the U.S., estimated new cases of colon cancer go up to 134,490, and 49,190 people will die in 2016 [2]. Today, six types of standard treatments are used to fight colon cancer: surgery, radiofrequency ablation, cryosurgery, chemotherapy, radiation therapy, and targeted therapy [3]. Chemotherapy is used before surgery to shrink the tumor, after surgery, or both before and after for patients with advanced stage cancer. A huge range of mutations such as chromosomal translocations, amplifications, and deletions can induce cancer development. If tumors are diagnosed with identical histopathology, mutations in each cancer can be different [4, 5]. Therefore, all therapeutic strategies, including chemotherapy, designed to target individual signaling molecules have limitations in improving current survival rates. Thus, the effectiveness of chemotherapeutic agents is not uniform among patients, and the discovery of biological factors and novel therapeutic strategies is very important for colon cancer treatment.

microRNAs (miRNAs) are small, endogenous, single-stranded RNAs of 18–22 nucleotides in length, that are emerging as important modulators of gene expression. miRNAs participate in diverse biological processes such as cell differentiation, proliferation, and apoptosis through a myriad of targets [6]. Recent studies have provided several insights into the relationship between miRNAs and colon cancer, and miRNAs are potentially crucial for successful colon cancer therapy [7, 8].

Antimicrobial peptides (AMPs) play important roles in the innate immune system of all forms of life [9]. According to the AMP database (http://aps.unmc.edu/AP/main.php), over 2700 such peptides have been reported with more than 190 peptides listed as anti-cancer peptides. We previously reported that FF/CAP18, the analogue peptide of human cathelicidin AMP, can induce apoptotic cell death on SAS-H1 squamous cells carcinoma-derived cell line [10] and HCT116 cells colon cancer-derived cell line [11]. Although the relationships between several cancers and AMPs have been widely evaluated, there are many limitations in applying AMPs as new strategies for cancer therapy, such as digestive system complications and production costs. Therefore, investigating the mechanisms of AMPs effects against colon cancer from a wider perspective is needed to obtain novel therapeutic targets and strategies.

These observations triggered our interest to investigate the regulation and effects of miRNAs expression by AMPs in colon cancer cells. Here, we report that miR-663a is upregulated by the administration of human cathelicidin LL-37 and its analogue peptide FF/CAP18 in the colon cancer cell line HCT116. Our findings suggest that miR-663a modulates HCT116 cells by modulating cell proliferation and apoptosis, suppressing the chemokine receptor CXCR4, abrogating phosphorylation of Akt, and arresting the cell cycle at the G2/M phase via activation of p21. These results suggest that the induction of miR-663a and the consequent modulation of the CXCR4-p21 pathway could be targeted by AMPs for cancer treatment.

## Methods

### Cell line and reagents

Dr. Bert Vogelstein (Johns Hopkins University, Baltimore, MD, USA) provided the human HCT116 colon carcinoma-derived cell line. The cells were maintained in Dulbecco’s modified Eagle medium (Nacalai Tesque, Kyoto, Japan) containing 10% fetal bovine serum (Invitrogen, Carlsbad, CA, USA) and a 5% antibiotic/antimycotic mixed stock solution (Nacalai Tesque) at 37 °C and 5% CO_2_. Before being used for the experiments, cells were routinely maintained under exponential-proliferation conditions. The cells were treated with a 0.25% trypsin-EDTA solution (Nacalai Tesque) to dislodge them at each passage.

The primary structure of LL-37 in single amino acid code is as follows: LLGDFFRKSKEKIGKEFKRIVQRIKDFLRNLVPRTES. To enhance the antimicrobial activity, FF/CAP18 was designed by the replacement of a glutamic acid residue and a lysine residue with phenylalanine at positions 11 and 20, respectively, of the 27mer (FRKSKEKIGKEFKRIVQRIKDFLRNLV), which resulted from the removal of the first and last five amino acids of LL-37 [12]. FF/CAP18 (FRKSKEKIGKFFKRIVQRIFDFLRNLV) and its scrambled control peptide (Sc/FF: IKLIRFRGDVKQRFIKLERSFNKFFKV) were synthesized by the method previously described [10].

### miRNA micro array

HCT116 cells were seeded at a density of 5.0 × 10^5^ cells/well in a 6-well plate. After 24 h of incubation, cells were treated with LL-37 (40 μg/mL and 80 μg/mL) and FF/CAP18 (10 μg/mL and 40 μg/mL) for 24 h. Total RNA, including miRNA, was extracted using the miRNeasy Mini Kit (QIAGEN, Venlo, The Netherlands) according to the manufacturer’s instructions. RNAs quality (concentrations, OD260/280 and OD260/230) were measured using a BioSpec-nano (Shimadzu, Kyoto, Japan), and 100 ng of each total RNA was analyzed. Labeling and hybridization were conducted using the miRNA Complete Labeling Reagent and Hyb kit (Agilent Technologies, Santa Clara, CA), and then scanned on an Agilent Technologies Microarray Scanner (Agilent Technologies). Each spot was quantified using Agilent Feature Extraction 10.7.3.1 (Agilent Technologies).

### RT-qPCR

To conduct the quantitative reverse transcription PCR (RT-qPCR) for the detection of miR-663a expression level, complementary DNA (cDNA) synthesis from miRNA was performed using miRCURY LNA™ Universal RT microRNA PCR Starter Kit (EXIQON, Vedbaek, Denmark), according to manufacturer’s instructions. Then, real-time PCR amplification was performed in a 10 μL solution containing PCR master mix, PCR primer mix (EXIQON, product no: 204284), and cDNA template. Similarly, cDNA synthesis from total RNA was carried out using PrimeScript™ II 1st strand cDNA Synthesis Kit (TaKaRa Bio, Shiga, Japan) and RT-PCR was performed in a 12.5 μL solution containing 2 × SYBR Premix Ex Taq II (Tli RNaseH Plus, TaKaRa Bio), 1 μL cDNA, and 0.4 μM of each primer. The sequences of the primers are available in Additional file 1: Table S1). Each RT-qPCR assay was performed in triplicate using the Thermal Cycler Dice Real Time System Single (TaKaRa Bio). Relative expression levels were calculated by the ΔΔCt method. The expression levels of target genes such as miR-663a and other genes were normalized to U6 and GAPDH, respectively.

### Establishment of miRNA over-expressing cells

To establish miR-663a over-expressing colon cancer cells, pLV-[hsa-mir-663a] plasmid (BioSettia, San Diego, CA) was introduced into the HCT116 cells. Recombinant lentiviruses with vesicular stomatitis virus G glycoprotein were produced as described previously [13]. HCT116 cells were seeded at a density of 1.0 × 10^5^ cells/well in a 6-well plate and inoculated with pLV-[hsa-mir-663a] lentivirus at a multiplicity of infection (MOI) of five for virus in the presence of 6 μg/mL polybrene. HCT116 cells with pLV-[hsa-mir-ctrl] (empty vector) were established as a control cell line in a similar manner (HCT116 miR-ctrl). Cells transduced with these vectors were cloned using 1 μg/mL puromycin administered for 48 h before each experiment.

### Proliferation assay

Cell proliferation was measured by WST-8 colorimetric assay (Dojindo, Tokyo, Japan). Cells were seeded in 96-well plates at a cell density of 2 × 10^3^ and incubated for 24 h. Cells were incubated with or without peptides for another 48 h at the final concentrations in the range of 1.56–100 μg/mL and the assay was conducted according to manufacturer’s instructions. WST-8 reagent was added to each well, and the samples were incubated for 4 h. The absorbance was measured at a wavelength of 450 nm using Synergy™ HT (BioTek, Winooski, VT, USA). All assays were performed in three independent experiments (*n* = 3). Cell viabilities (%) were calculated as relative values based on the absorbance of non-treated cells (100%).

### Cell cycle analysis

Cell cycle analysis was carried out using the Muse Cell Cycle Kit and Muse Cell Analyzer (Merck Millipore, Darmstadt, Germany). Cells were harvested by trypsinization and fixed by 70% ice-cold ethanol. After fixation, cell pellets were resuspended in Muse Cell Cycle Kit reagent, incubated for 30 min, and protected from light until further analysis. Cell cycle analysis carried out by the method supplied by the manufacturer.

### Western blot analysis

Cells were homogenized in lysis buffer (1 M Tris-HCl at pH 7.4, 3 M NaCl, 1% Triton X-100, 6 mM sodium deoxycholate, and 0.5% protease inhibitor cocktail (Nacalai Tesque, Kyoto, Japan). The detailed method for western blotting has been described in our previous report [14]. A mouse monoclonal antibody against p21 (1:1000, Medical & Biological Laboratories Co., LTD., Aichi, Japan), p53 (1:1000, Abcam, Cambridge, UK), cdc2 (1:1000, Cell Signaling Technology (CST), Danvers, MA, USA), and α-tubulin (1:1000, Santa Cruz Biotechnology, Inc., Dallas, US), and rabbit monoclonal antibodies against Akt (1:1000, CST), phospho-Akt (1:2000, CST), and phospho-cdc2 (1:1000, CST) were used as primary antibodies. The corresponding secondary antibodies: sheep anti-mouse IgG conjugated to horseradish peroxidase and sheep anti-rabbit IgG conjugated to horseradish peroxidase, 1:2000 (GE Healthcare, Little Chalfont, Buckinghamshire, UK) were used as secondary antibodies. Signals on the membranes were detected using an ECL prime kit (GE Healthcare), and the image was obtained with the LumiCube (Liponics, Inc., Tokyo, Japan). Signal intensity was quantified by using the JustTLC software (Sweday, Sodra Sandby, Sweden).

### Xenograft model

Animal experiments were conducted in accordance with the guideline for the Regulations for Animal Experiments and Related Activities at Tohoku University (Approval number: 2016AgA-039). The backs of 6-week- old female BALB/cA.Jcl-*nu*/*nu* (CLEA Japan Inc., Tokyo, Japan) were inoculated with 5.0 × 10^6^ HCT116 cells. Tumor size was monitored at 2-day intervals by measuring the length and width with calipers, and its volumes were calculated with the formula: (L × W^2^) × 0.5, where L is length and W is width of each tumor. FF/CAP18 and Sc/FF were co-administered at 10 mg/kg per mouse. Tumor weight was determined at day 14. Medetomidine hydrochloride (0.3 mg/kg), midazolam (4 mg/kg), and butorphanol tartrate (5 mg/mL) were administrated by intraperitoneal injection for anesthesia. Mice were sacrificed by cervical spine fracture dislocation and organs were collected for pathological examination.

### Statistical analysis

The data are expressed as the mean ± SD of three independent experiments performed in triplicate. The statistical analyses were performed using the Student’s *t* test. A *p*-value < 0.05 was considered statistically significant.

## Results

### miR-663a is a major upregulated miRNA in HCT116 cells treated with LL-37 and FF/CAP18

**Fig. 1:**
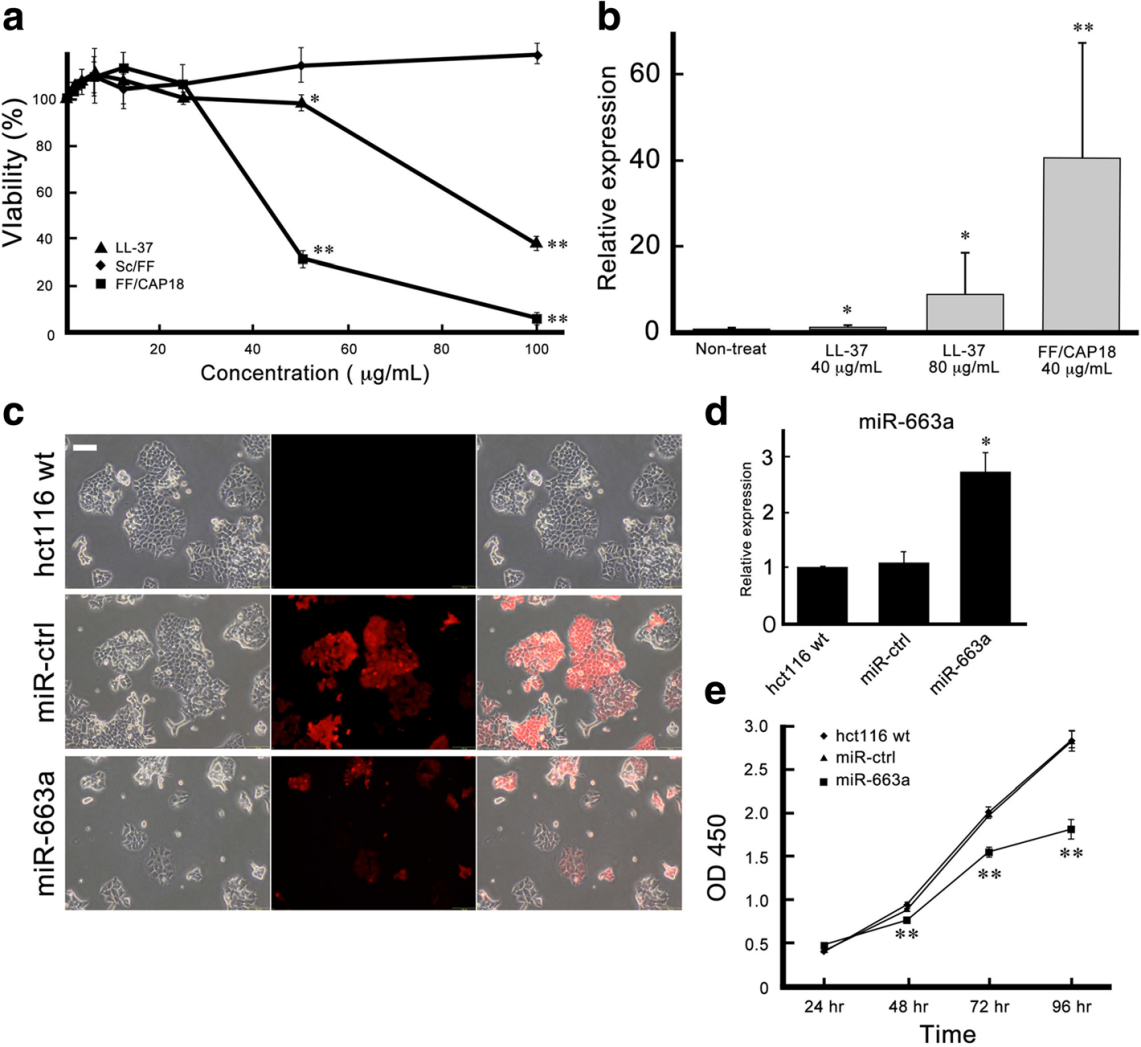
miR-663a is the major upregulated miRNA in HCT116 cells treated with LL-37 and FF/CAP18. **a** Viability of HCT116 cells after treatment with LL-37, FF/CAP18, or Sc/FF for 48 h at the concentration of 1.56–100 μg/mL. Each data is presented as mean ± SD of triplicate experiments. (* *p* < 0.05; ** *p* < 0.001). **b** Relative expression of miR-663a in HCT116 cells treated with LL-37 (40, 80 μg/mL) and FF/CAP18 (40 μg/mL). Each data is shown as mean ± SD of triplicate experiments. (* *p* < 0.05; ** *p* < 0.001). **c** Morphology of established over-expressing miR-663a HCT116 cells using the lentivirus vector system. HCT116 cells transduced with lentiviruses harboring control vector (miR-ctrl) and miR-663a-expressing vector (miR-663a) expressed red fluorescence (rPuro). **d** The RT-qPCR analysis showed that HCT116 cells transduced with miR-663a-expressing vector expressed miR-663a. Each data is shown as mean ± SD of triplicate experiments. (* *p* < 0.05). **e** Proliferation of HCT116 cells (wt) and HCT116 cells transduced with lentiviruses harboring control vector (miR-ctrl) and miR-663a-expressing vector (miR-663a) for 4 days. Each data is presented as mean ± SD of triplicate experiments. (** *p* < 0.001)

The cathelicidin antimicrobial peptide, LL-37, induced growth suppression significantly in the colon cancer cell line HCT116 (Fig. 1a) at a concentration higher than 50 μg/mL. FF/CAP18, an analogue peptide of LL-37, suppressed growth more intensely compared to LL-37. We attempted to reveal the behavior of miRNA in HCT116 cells during the growth suppression induced by the AMPs. miRNA array analysis revealed that 17 miRNAs were increased 2-fold or greater in HCT116 cells after treatment with LL-37 (40 μg/mL) compared to that in non-treated cells (Table 1). Among these miRNAs, miR-663a and miR-513a-5p were upregulated dose-dependently after the administration of LL-37. Both miRNAs showed higher expression levels in HCT116 cells treated with FF/CAP18 than those treated with LL-37. The expression level of miR-663a increased 80 times or greater compared with miR-513a-5p. This result confirmed reproducibility in other RT-qPCR experiments (Fig. 1b). These results prompted us to explore the role of miR-663a in HCT116 cells.

**Table 1:** Upregulated miRNAs in HCT116 treated with AMPs

	Fold Change		
	LL-37		FF/CAP18
miRNA	40 mg/mL	80 mg/mL	40 mg/mL
hsa-miR-3663-3p	162.04	ND	58.19
hsa-miR-4271	145.10	ND	359.22
hsa-miR-630	139.54	ND	194.83
hsa-miR-371a-5p	123.04	ND	ND
hsa-miR-1181	112.97	ND	ND
hsa-miR-575	112.85	ND	315.56
hsa-miR-939-5p	96.13	ND	83.80
hsv2-miR-H6-5p	94.15	ND	ND
hsa-miR-663a	61.85	215.81	277.49
hsa-miR-135a-3p	27.81	ND	ND
hsa-miR-132-3p	3.21	ND	ND
kshv-miR-K12-3-5p	3.16	3.09	ND
hsa-miR-10a-5p	3.05	ND	ND
hsa-miR-762	3.03	2.16	ND
hsa-miR-513a-5p	2.26	4.59	8.37
hsa-miR-99a-5p	2.10	ND	ND
hsa-miR-1915-3p	2.09	2.41	ND

*ND* Not detectable

### Over-expression of miR-663a delays cell proliferation in HCT116 cells

To identify the role of miR-663a in HCT116 cells, we established over-expressing miR-663a HCT116 cells using a lentivirus vector system. HCT116 cells transduced with lentiviruses harboring control vector (Fig. 1c: miR-ctrl cells) and miR-663a-expressing vector (Fig. 1c: miR-663a cells) expressed red fluorescence (Fig. 1c: rPuro). The RT-qPCR identified that HCT116 cells transduced with miR-663a-expressing vector expressed miR-663a 2–3-folds higher than control vector-introduced HCT116 cells (Fig.1d). In miR-663a overexpressing cells, colony morphology was smaller than non-infected cells (wt) and control cells (Fig. 1c). Moreover, miR-663a over expressing cells exhibited senescence-like morphology displayed as enlarged cytosol (Fig. 1c). These morphological features motivated us to examine the proliferation, and the WST-8 assay revealed that miR-663a expressing cells had suppressed growth compared to HCT116 cells and miR-ctrl cells (Fig. 1e). Thus, these results suggest that miR-663a is the main upregulated miRNA stimulated by the antimicrobial peptides LL-37 and FF/CAP18 and its expression has an anti-proliferative effect on colon cancer cells.

### Anti-proliferative effect of miR-663a is through p53-independent p21 phosphorylation

**Fig. 2:**
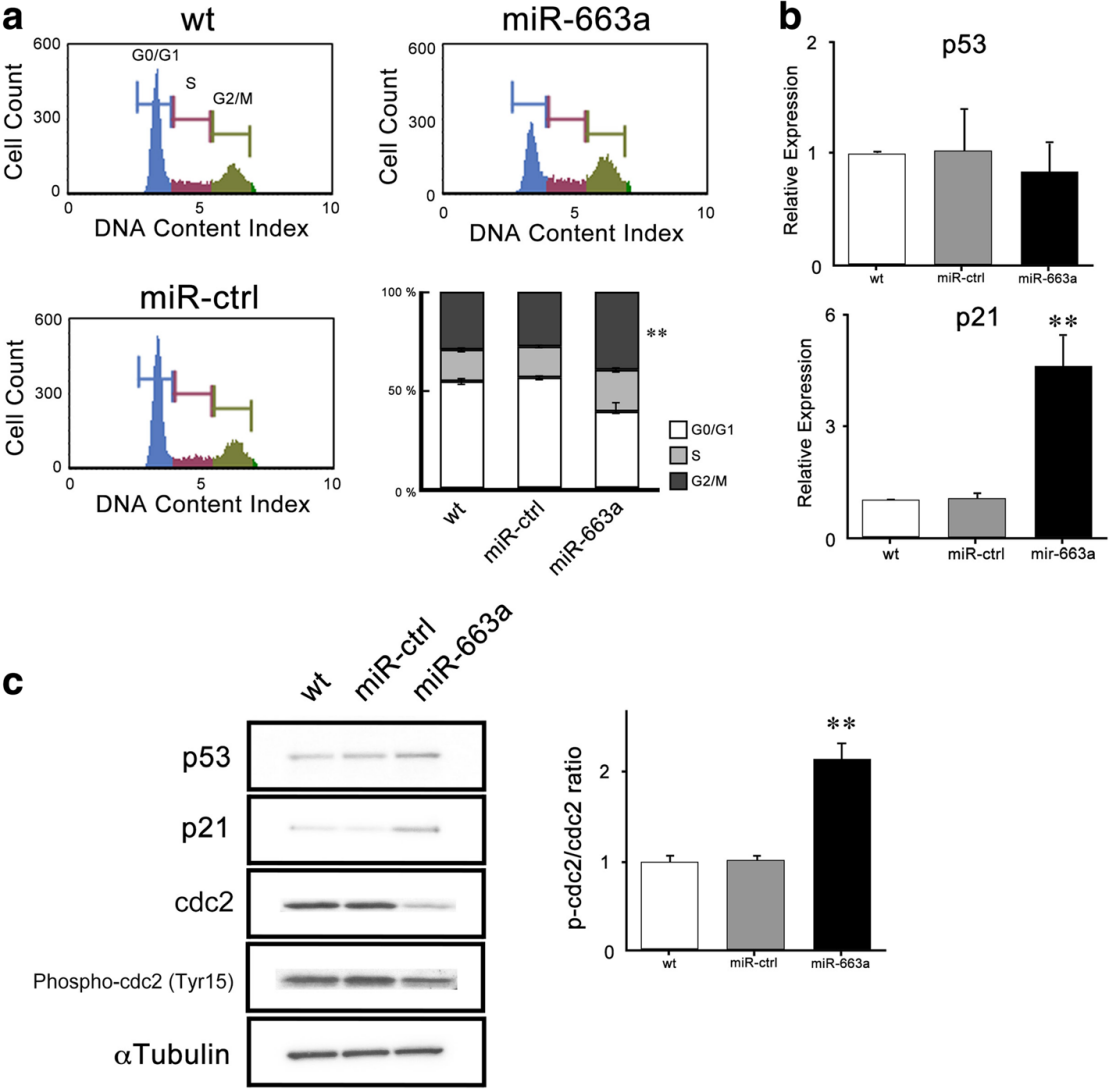
miR-663a induced cell cycle arrest following p21 expression and accumulation of the inactive form of cdc2 in HCT116 cells. **a** Cell cycle was examined by the MUSE cell analyzer and representative data are shown. The percentage of cells in G0/G1, S, and G2/M phases are presented as mean ± SD of triplicate experiments. (** *p* < 0.001). **b** The mRNA expression of p21 and p53 were determined by qPCR and relative expression levels are shown as mean ± SD of triplicate experiments. (** *p* < 0.001). **c** Protein levels of p53, p21, cdc2, phospho-cdc2 (Tyr15), and αtubulin in the total cell lysates were determined by western blotting analysis. Representative data of three experiments are shown. Ratio of phospho-cdc2 (Tyr15)/cdc2 was calculated after digitalization by using JustTLC and shown as mean ± SD of triplicate experiments. (** *p* < 0.001)

We sought to reveal the mechanisms of the anti-proliferative effect on HCT116 cells due to the upregulation of miR-663a. Cell cycle analysis uncovered that miR-663a over-expressing cells are arrested in the G2/M phase compared with wt and miR-ctrl cells, whereas cells in G1/G0 phase are decreased (Fig. 2a). Cell cycle is regulated in a rigorous manner by various regulators. The p53 gene, called ‘the guardian of the genome,’ is one of the most important genes for control of the cell cycle and cell death [15]. This gene expression level was not changed between the three types of HCT116 cells (Fig. 2b, upper). Interestingly, p21, the downstream transcription target gene of p53, was upregulated in over-expressing miR-663a HCT116 cells (Fig. 2b, lower). These tendencies were also confirmed at protein levels (Fig. 2c). Moreover, we confirmed expression levels of the cell cycle regulators involved in the G2/M phase, total cdc2 protein, and cdc2 phosphorylated at tyrosine (Tyr) 15. Western blotting revealed that the total cdc2 level in HCT116 miR-663a was lower than that in wt and miR-ctrl, and the ratio of phospho-cdc2 (Tyr 15) /total cdc2 (p-cdc2/cdc2) was higher than that in other two types of HCT116 cells (Fig. 2c). These observations indicate that miR-663a could cause cell cycle arrest in the G2/M phase in colon cancer cells mostly through a p21 dependent mechanism.

### Over-expressing miR-663a suppresses tumorigenesis in a mouse xenograft model

**Fig. 3:**
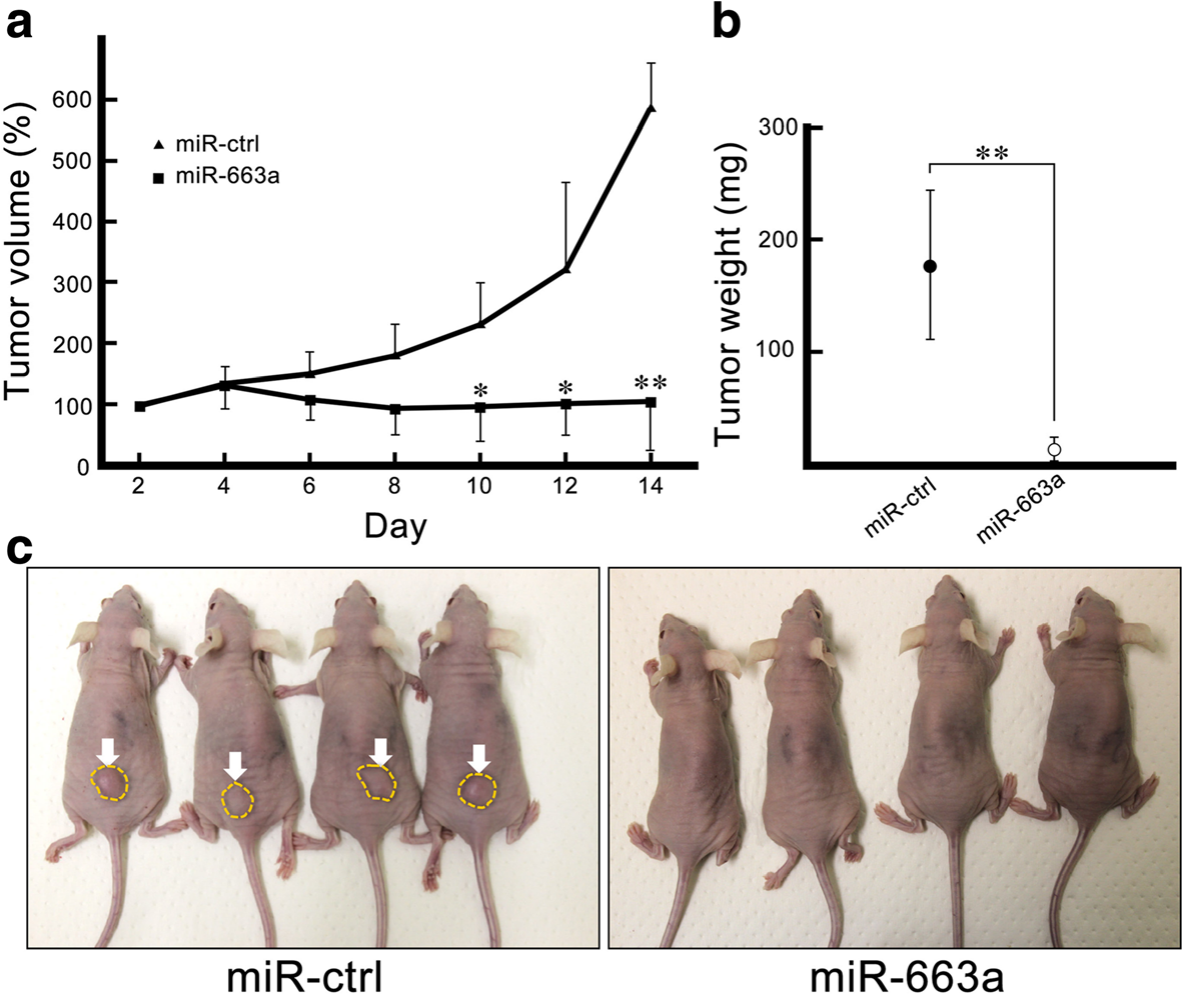
miR-663a suppresses tumorigenesis of HCT116 in a xenograft model. **a** Growth curves of HCT116 tumors after nude mice were injected with miR-663a (square) or control miRNA (triangle). The volume of the tumors derived from both cells was evaluated at 2-day intervals for 14 days and plotted as the percentage relative to day 0. Each plot is shown as mean ± SD of four experiments (* *p* < 0.05; ** *p* < 0.001). **b** Tumor weight was measured 14 days after the inoculation of over-expressing miR-663a HCT116 cells harboring control vector. Each plot is shown as mean ± SD of four experiments (** *p* < 0.001). **c** Photographs illustrating mice tumors derived from control cells (miR-ctrl: arrows) and over-expressing miR-663a HCT116 cells (miR-663a) 14 days after inoculation

To evaluate the possible anti-tumorigenic effects of miR-663a, we performed a xenograft model assay. Average tumor volume between the group inoculated with HCT116 miR-ctrl and HCT116 miR-663a was significantly different between 10 and 14 days (Fig. 3a). The tumor weight of the miR-663a group at 14 days was also lighter than that of the miR-ctrl group (Fig. 3b). We examined whether FF/CAP18 has anti-tumorigenesis effects in HCT116 cells. Tumor volume and final weight were reduced significantly in the group of mice simultaneously inoculated with HCT116 cells and FF/CAP18 (10 mg/kg) compared with the Sc/FF (10 mg/kg) inoculation group (Additional file 2: Figure S1). Histopathologically, subcutaneous tumor cells were similar to each other in the xenograft model. There was no translocation to various organs such as the kidney, spleen, heart, or liver, with only one exception of the lung in the ctrl group (data not shown). These results indicate that introduction of miR-663a suppresses the growth of human colon cancer cells in tumors in vivo and in vitro. Additionally, miR-663a can contribute in inhibiting tumorigenesis of HCT116 cells treated with FF/CAP18.

### Anti-proliferative effects arising from miR-663a are executed through the CXCR4-Akt pathway

**Fig. 4:**
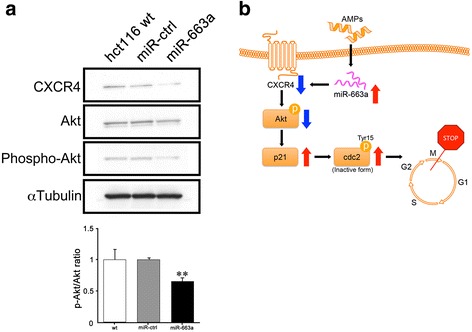
miR-663a can target the CXCR4-Akt pathway. **a** Protein levels of CXCR4, Akt, phospho-Akt, and αtubulin in the total cell lysates were determined by western blotting analysis. Representative data is shown in triplicate experiments. Ratio of phospho-Akt/Akt was calculated after digitalization by using JustTLC and shown as mean ± SD of triplicate experiments (** *p* < 0.001). **b** Putative model of anti-cancer effect of AMPs in HCT116 cells. miR-663a upregulated by AMPs suppresses CXCR4 expression leading to reduced phosphorylation of Akt. Consequently, p21 and the inactive form of cdc2 accumulation are caused, which results in the cell cycle arrest at the G2/M phase

Results so far demonstrate that miR-663a could induce anti-proliferative effects on colon cancer cells via p21-associated cell cycle arrest in the p53 independent pathway. To identify the detailed mechanism of this pathway, we focused on Akt and CXCR4. Western blotting detected that phospho-Akt and CXCR4 expressions were decreased in HCT116 miR-663a compared to HCT116 wt and miR-ctrl cells (Fig. 4a).

## Discussion

miRNAs play a key role in many crucial biological processes such as cell proliferation, differentiation, and apoptosis in colon cancer, and continued efforts will help identify new targets for the diagnosis, prognosis, and management of colon cancer [8, 16, 17]. Recently, miR-663a, which belongs to the primate specific miRNAs, was detected as a tumor suppressing miRNA in several cancer cells including colon cancer cells [18–20]. In the present study, we identified miR-663a as the upregulated miRNA in the colon cancer cell line HCT116 treated with human cathelicidin AMP, LL-37 and its analogue, FF/CAP18. Moreover, we revealed that upregulated miR-663a in HCT116 cells induced the suppression of growth through cell cycle arrest at the G2/M phase.

LL-37 is the C-terminal domain of hCAP18 protein and known as the only member of the cathelicidin family AMPs in human. LL-37 is cleaved by the protease from hCAP18 and shows various effects other than antibacterial activity including immune regulation [21, 22]. LL-37 is expressed in the epithelial cells of numerous organs [23], and is increasingly recognized as a novel modulator of tumor growth and metastasis in carcinogenesis of various cancers [24]. Ren et al. has revealed that LL-37 expression was significantly suppressed in human colon cancer tissue compared with normal tissue, and LL-37 can induce caspase-independent apoptosis in colon cancer cells. Furthermore, in this report, CRAMP, a mouse cathelicidin AMP, deficient mice demonstrate high ratio of carcinogenesis induced by azoxymethane administrations [25]. However, it has been reported that LL-37 is expressed in human lung cancer cells (20–30 ng/mL) and acts as a growth factor [26]. In this report, however, the concentration of LL-37 necessary to activate lung cancer cell proliferation was in the order of ng/mL, whereas the administration of 20 μg/mL LL-37 decreased the cell numbers. In prostate cancer and ovarian cancer, LL-37 is also overexpressed and associated with proliferation [27, 28]. These observations suggest that the role of LL-37 in each cancer is different and its control is surprisingly complicated, which results in one of the limitations of its application in cancer treatment. The relationship between AMPs and cancer is not discussed with regard to miRNAs to date, thus this study is the first report. As previously described, miR-663a has been reported to be associated with cancer suppression in several cancers, although its increased level was found in lung cancer, ovarian cancer [29, 30], and castration-resistant prostate cancer [31]. These observations indicate that the differential sensitivity of LL-37 among cancer types is associated with the expression of miR-663a, although further investigation is needed.

C-X-C chemokine receptor type 4 (CXCR4) is broadly expressed in various malignant tumors including colon cancer [32]. CXCR4 is upregulated by the microenvironment, and isolated metastatic cells likely require CXCR4 signals to initiate their proliferation, suggesting that CXCR4 inhibitors have potential as anticancer agents to suppress the outgrowth of micrometastasis [33]. The present study shows that CXCR4 expression was reduced in over-expressing miR-663a HCT116 cells, which resulted in the suppression of proliferation. Yu et al. reports that miR-663a negatively regulates CXCR4 expression by targeting its coding sequence in human glioblastoma cells and compromises proliferation [34]. This report suggests that miR-663a directly binds to the coding sequence of CXCR4 mRNA to suppress its translation. Thus, the suppression of proliferation in over-expressing miR-663a colon cancer cells might be induced by the same mechanism as in glioblastoma cells. We confirm down-regulation of CXCR4 protein expression and upregulation of the p-cdc2/cdc2﻿ ratio in HCT116 cells treated with FF/CAP18 (Additional file 3: Figure S2), which demonstrates that one of the pathways of the anti-cancer mechanism of AMPs can be triggered by targeting CXCR4 via miR-663a overexpression and changing the downstream signals containing p21 and cdc2 (Fig. 4b).

From previous reports, the mechanism of the anti-cancer effects of AMPs against cancer cells includes multiple directions such as apoptosis, necrosis, and autophagy [35–37]. We previously reported that FF/CAP18 induces apoptotic cell death in HCT116 cells through dynamic changes in the levels of metabolic profiles and revealed that the glycolytic pathway was restricted, resulting in reduced energy status [38]. To reveal the relationship between miR-663a and metabolism in HCT116 cells, we have performed metabolome analysis in a similar way. We could not observe appreciable changes in the glycolytic pathway and energy status (Additional file 4: Figure S3). Furthermore, we confirmed that LL-37 and FF/CAP18 induce anti-proliferative effect on another colon cancer cell line, Caco2, which was accompanied by the upregulation of miR-663a expression as well as on HCT116 cells (Additional file 5: Figure S4). These data support previous hypotheses that AMPs can act against cancer, including colon cancer, through a variety of mechanisms of action, and the modulation of miR-663a could be just one factor inducing the negative effect on cancer by the administration of AMPs.

We showed that miR-663a inhibits the growth of colon cancer cells in vitro and in vivo by upregulating p21 gene expression and the subsequent induction of cell cycle arrest and apoptosis. Similar result has been reported about miR-6734 in the colon cancer cell HCT116 [39]. In this study, miRNA microarray identified 17 miRNAs, including miR-663a, as upregulated miRNAs in HCT116. However, miR-6734 was not upregulated. Thus, the regulation system of cancer growth can include various signal controls via miRNA.

Recently, new drug delivery systems have been studied to improve AMP’s activities and overcome transportation problems. For instance, theranostics and drug delivery strategies using magnetic anti-cancer drugs are expected to bring new hope for cancer therapy [40]. Indeed, Niemirowicz et al. reported that magnetic nanoparticles enhance the anticancer activity of LL-37 against colon cancer cells [41]. Moreover, using mesoporous silica nanoparticles has been investigated [42]. Therefore, AMPs, including LL-37 and its modified peptides, have the potential to be anticancer agents if they are appropriately targeted using these drug delivery systems.

## Conclusions

We report that human cathelicidin antimicrobial peptide, LL-37, and its analogues suppress the proliferation of colon cancer cells via the upregulation of miR-663a abrogating CXCR4 expression. This study contributes to the understanding of anti-cancer mechanisms in colon cancer cells treated with AMPs, and highlights the possibility of using AMPs for future strategies in cancer therapy.

## Additional files

Additional file 1: Table S1. List of primers. (DOCX 40 kb)
Additional file 2: Figure S1. FF/CAP18 suppresses tumorigenesis of HCT116 in a xenograft model. (A) Growth curves of HCT116 tumors after simultaneous injection of nude mice with FF/CAP18 (square) or control peptide, Sc/FF (triangle). The volume of the tumors was derived from both cells evaluated at 2-day intervals for 14 days and plotted as the percentage relative to day 0. Each plot is shown as mean ± SD of four experiments (* *p* < 0.05; ** *p* < 0.001). (B) Tumor weight was measured 14 days after inoculation of HCT116 cells with Sc/FF and FF/CAP18. Each plot is shown as mean ± SD of four experiments (* *p* < 0.05). (C) Photographs illustrating mice tumors derived from control cells treated with Sc/FF (Sc/FF: arrows) and HCT116 cells treated with FF/CAP18 (FF/CAP18) 14 days after inoculation. (JPG 379 kb)
Additional file 3: Figure S2. Suppression of CXCR4 and phosphorylation of cdc2 are observed in HCT116 cells treated with FF/CAP18. (A) Protein levels of CXCR4, cdc2, phospho-cdc2, and α-tubulin in the total cell lysate were determined by western blotting analysis. Representative data are shown in triplicate experiments. Ratio of phospho-cdc2/cdc2 was calculated after digitalization by using JustTLC and shown as mean ± SD of triplicate experiments. (** *p* < 0.001). (JPG 296 kb)
Additional file 4: Figure S3. Metabolome data map of the glycolysis metabolic pathway in HCT116 cells. Each bar represents the relative amount of a metabolite for HCT116 (blue) transduced with control vector (red) or miR-663a over-expressing vector (green). All metabolite data are shown as the mean of triplicate samples ± SD. Detailed materials and methods are shown in our previous manuscript [38]. (JPG 522 kb)
Additional file 5: Figure S4. miR-663a is upregulated in Caco2 cells treated with LL-37 and FF/CAP18 the same as in HCT116 cells. (A) Viability of Caco2 cells after treatment with LL-37, FF/CAP18, or Sc/FF for 48 h at the concentration of 100–1.5625 μg/mL. Each data is presented as mean ± SD of triplicate experiments. (* *p* < 0.05; ** *p* < 0.001). Caco2 cells were purchased from RIKEN Bioresource Center (RBRC-RCB0988) and maintained in a same condition as HCT116 cells. (B) Relative expression of miR-663a in Caco2 cells treated with LL-37 (40, 80 μg/mL) and FF/CAP18 (40 μg/mL). Each data is shown as mean ± SD of triplicate experiments. (* *p* < 0.05; ** *p* < 0.001). (JPG 469 kb)

## Abbreviations

AMPs: Antimicrobial peptides
CXCR4: C-X-C chemokine receptor type 4
hCAP: Human cathelicidin antimicrobial peptide
