# Supplementary material for: miR-663a regulates growth of colon cancer cells, after administration of antimicrobial peptides, by targeting CXCR4-p21 pathway
Source: BMC Cancer. 2017 Jan 7;17:33. doi: 10.1186/s12885-016-3003-9 (PMC5219750; doi:10.1186/s12885-016-3003-9)
Supplement: Additional file 1: Table S1. — List of primers. (DOCX 40 kb) [file 12885_2016_3003_MOESM1_ESM.docx]

Table S1. List of primers.

| Gene name | Forward (5' - 3') | Reverse (5' - 3') |
| --- | --- | --- |
| U6 | CTCGCTTCGGCAGCACA | AACGCTTCACGAATTTGCGT |
| p53 | ATGGAGGAGCCGCAGTCAGAT | GCAGCGCCTCACAACCTCCGT |
| p21 | AGGCACCGAGGCACTCAGAG | AGTGGTAGAAATCTGTCATGCTG |
| GAPDH | AAGGTGAAGGTCGGAGTCAAC | GGGGTCATTGATGGCAACAATA |
