# Supplementary figures and images for: miR-663a regulates growth of colon cancer cells, after administration of antimicrobial peptides, by targeting CXCR4-p21 pathway
Source: BMC Cancer. 2017 Jan 7;17:33. doi: 10.1186/s12885-016-3003-9 (PMC5219750; doi:10.1186/s12885-016-3003-9)

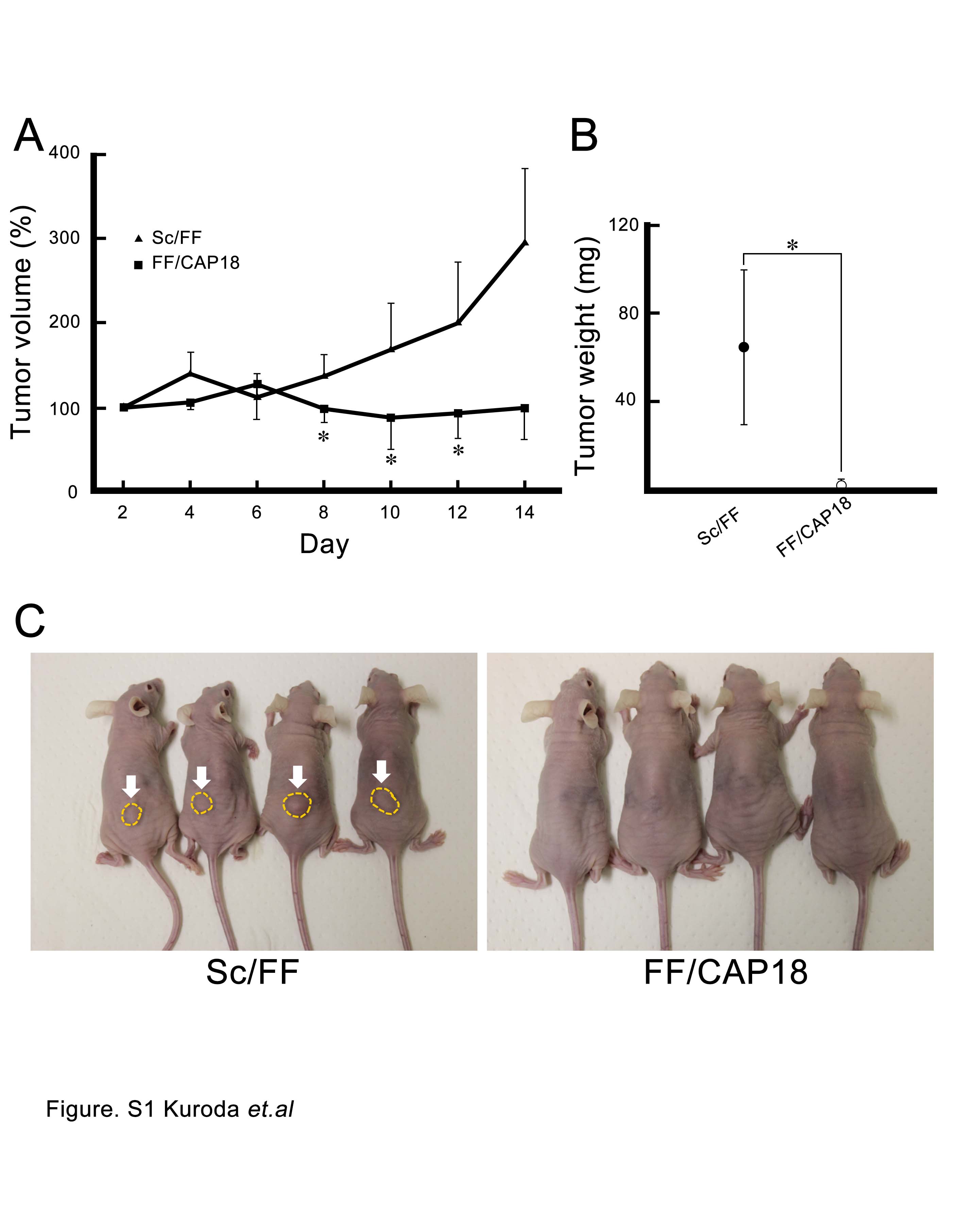

Supplement: Additional file 2: Figure S1. — FF/CAP18 suppresses tumorigenesis of HCT116 in a xenograft model. (A) Growth curves of HCT116 tumors after simultaneous injection of nude mice with FF/CAP18 (square) or control peptide, Sc/FF (triangle). The volume of the tumors was derived from both cells evaluated at 2-day intervals for 14 days and plotted as the percentage relative to day 0. Each plot is shown as mean ± SD of four experiments (* p < 0.05; ** p < 0.001). (B) Tumor weight was measured 14 days after inoculation of HCT116 cells with Sc/FF and FF/CAP18. Each plot is shown as mean ± SD of four experiments (* p < 0.05). (C) Photographs illustrating mice tumors derived from control cells treated with Sc/FF (Sc/FF: arrows) and HCT116 cells treated with FF/CAP18 (FF/CAP18) 14 days after inoculation. (JPG 379 kb) [file 12885_2016_3003_MOESM2_ESM.jpg]

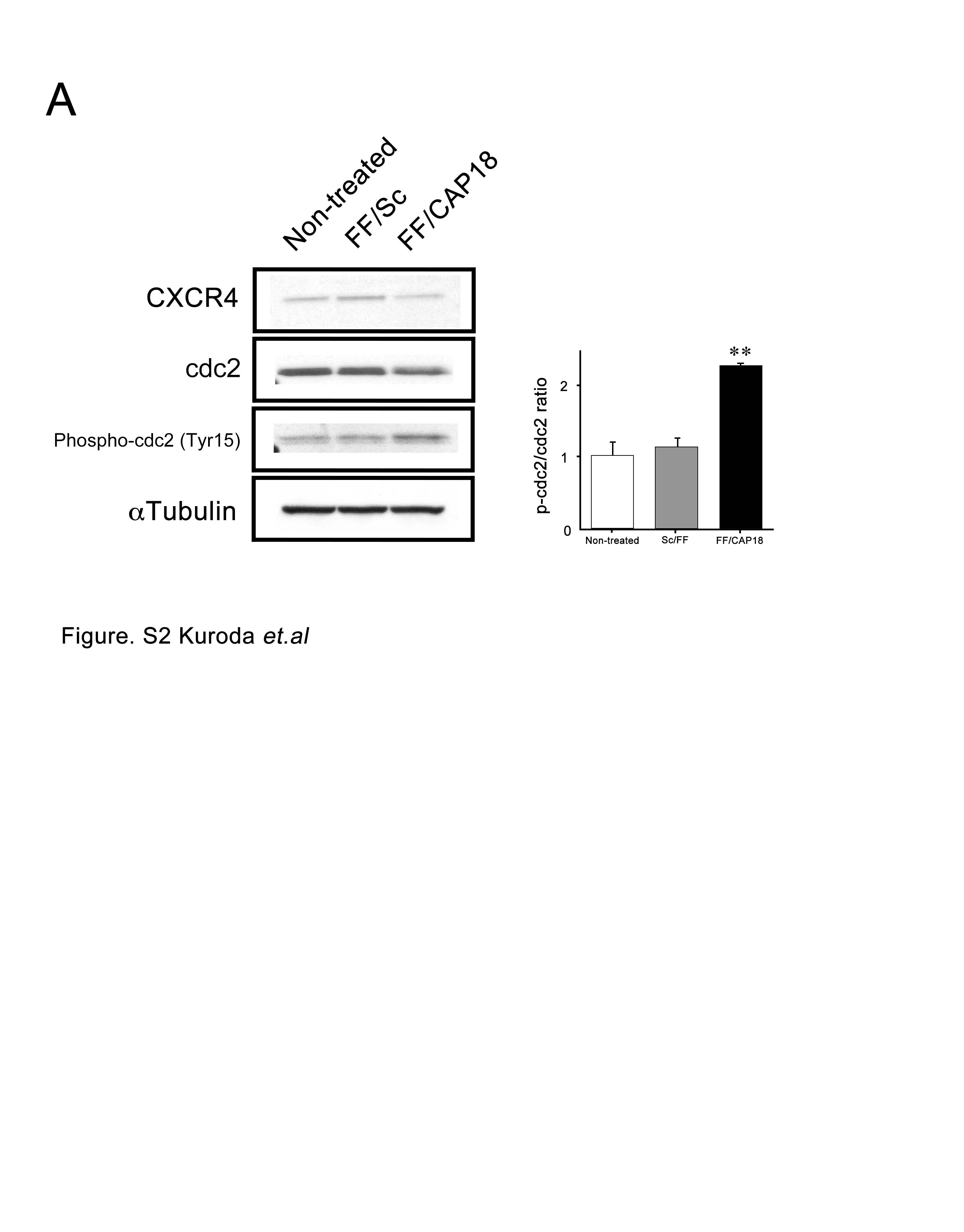

Supplement: Additional file 3: Figure S2. — Suppression of CXCR4 and phosphorylation of cdc2 are observed in HCT116 cells treated with FF/CAP18. (A) Protein levels of CXCR4, cdc2, phospho-cdc2, and α-tubulin in the total cell lysate were determined by western blotting analysis. Representative data are shown in triplicate experiments. Ratio of phospho-cdc2/cdc2 was calculated after digitalization by using JustTLC and shown as mean ± SD of triplicate experiments. (** p < 0.001). (JPG 296 kb) [file 12885_2016_3003_MOESM3_ESM.jpg]

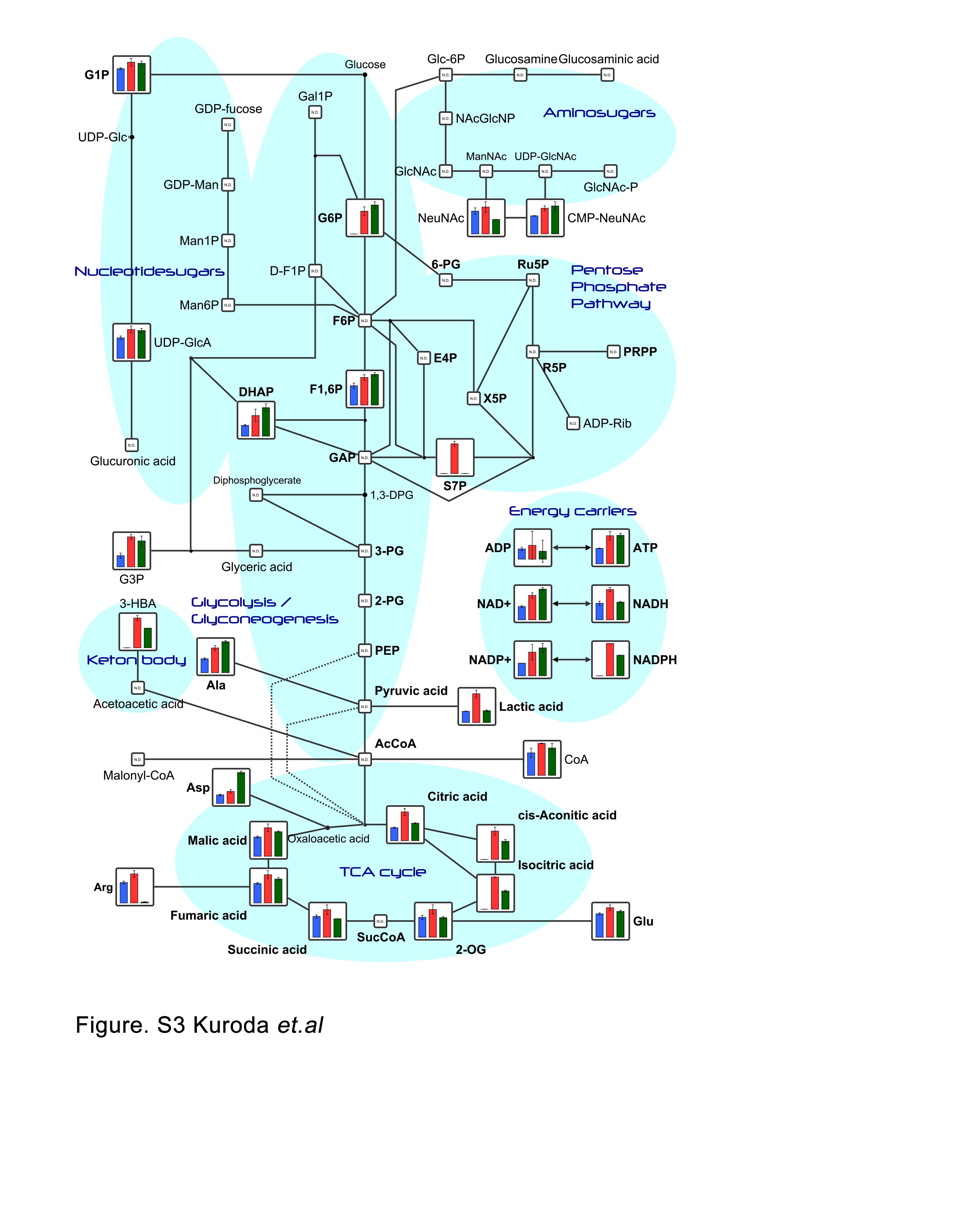

Supplement: Additional file 4: Figure S3. — Metabolome data map of the glycolysis metabolic pathway in HCT116 cells. Each bar represents the relative amount of a metabolite for HCT116 (blue) transduced with control vector (red) or miR-663a over-expressing vector (green). All metabolite data are shown as the mean of triplicate samples ± SD. Detailed materials and methods are shown in our previous manuscript [38]. (JPG 522 kb) [file 12885_2016_3003_MOESM4_ESM.jpg]

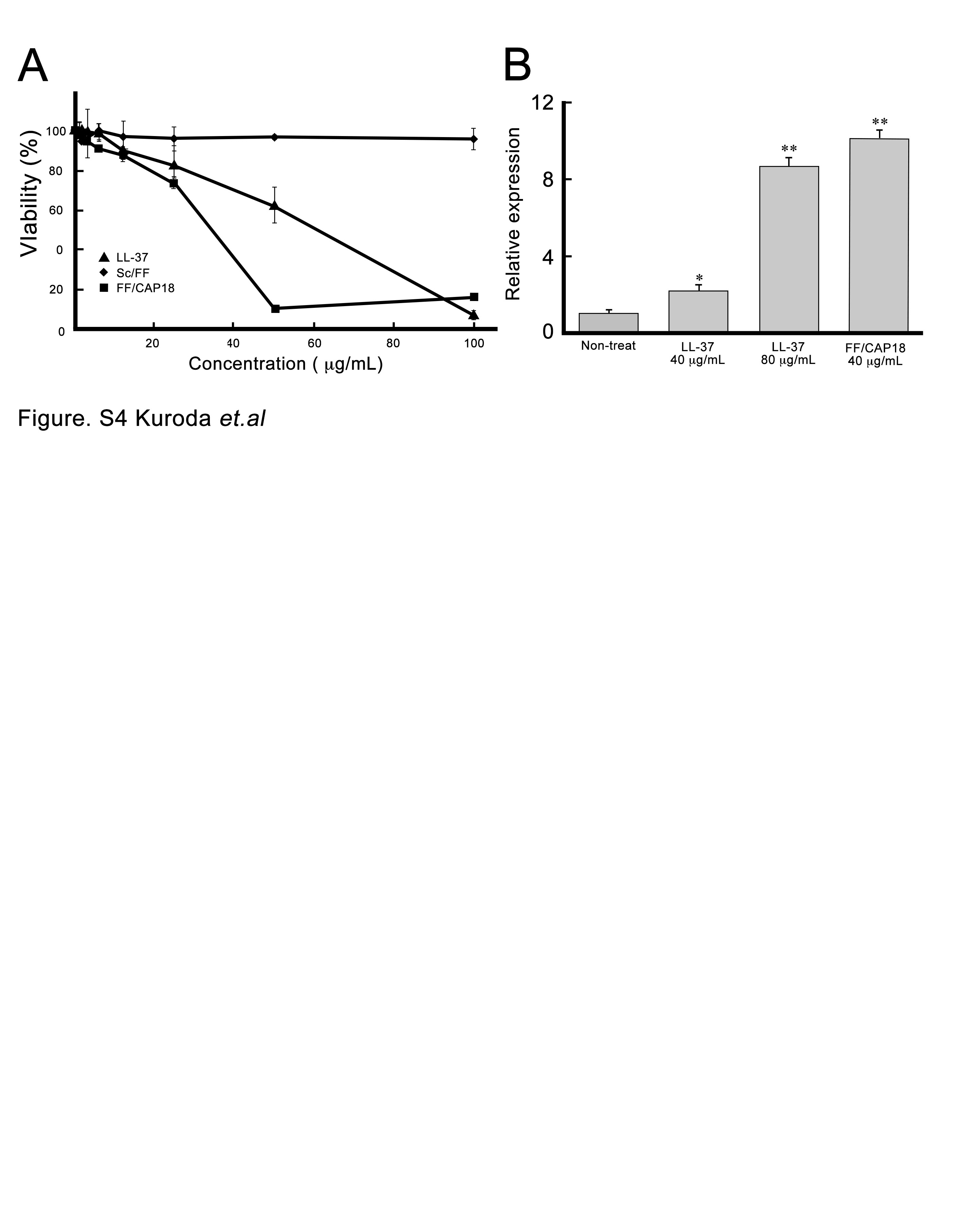

Supplement: Additional file 5: Figure S4. — miR-663a is upregulated in Caco2 cells treated with LL-37 and FF/CAP18 the same as in HCT116 cells. (A) Viability of Caco2 cells after treatment with LL-37, FF/CAP18, or Sc/FF for 48 h at the concentration of 100–1.5625 μg/mL. Each data is presented as mean ± SD of triplicate experiments. (* p < 0.05; ** p < 0.001). Caco2 cells were purchased from RIKEN Bioresource Center (RBRC-RCB0988) and maintained in a same condition as HCT116 cells. (B) Relative expression of miR-663a in Caco2 cells treated with LL-37 (40, 80 μg/mL) and FF/CAP18 (40 μg/mL). Each data is shown as mean ± SD of triplicate experiments. (* p < 0.05; ** p < 0.001). (JPG 469 kb) [file 12885_2016_3003_MOESM5_ESM.jpg]
